# Artificial intelligence in the mass production of natural enemies for biological control in modern agriculture

**DOI:** 10.1002/ps.70116

**Published:** 2025-08-14

**Authors:** Khadija Javed, Guy Smagghe, Ying‐Qian Kang, Qi Wang, Yong Wang

**Affiliations:** ^1^ College of Agriculture, Guizhou University Guiyang China; ^2^ Julius Kühn‐Institut (JKI) for Biological Control Darmstadt Germany; ^3^ Key Laboratory of Medical Microbiology and Parasitology, School of Basic Medical Sciences Guizhou Medical University Guiyang China; ^4^ Institute of Entomology, Guizhou University Guiyang China; ^5^ Department of Biology Vrije Universiteit Brussels (VUB) Brussels Belgium; ^6^ Key Laboratory of Environmental Pollution Monitoring and Disease Control Ministry of Education of Guizhou Guiyang China; ^7^ State Key Laboratory of Public Big Data, School of Computer Science and Technology, Guizhou University Guiyang China

**Keywords:** artificial intelligence, natural enemies, biological control, crop protection, sustainable agriculture, machine learning, computer vision, unmanned aerial vehicles, digital literacy, Artifical Neural Networks, convolutional neural networks, Deep Learning, Internet of Things

## Abstract

The increasing challenges posed by pest infestations in contemporary agriculture demand sustainable alternatives to conventional pesticides. Biological control (BC), which utilizes natural enemies (NEs) such as predators and parasitoids, offers an eco‐friendly approach to pest management. However, large‐scale production of NEs faces significant challenges, including high costs, time‐consuming processes, and inconsistent quality. Artificial intelligence (AI) is increasingly being applied in modern agriculture to enhance the efficient mass production of NEs for BC. By leveraging automation, machine learning (ML), computer vision, and real‐time data analytics, AI can improve key aspects of NEs production, including diet formulation, environmental control, behavioral monitoring, and quality assurance. AI‐driven systems promote consistency and scalability in NE manufacturing, while adaptive feedback mechanisms enable continuous process optimization. Furthermore, AI supports the development of predictive models and customized distribution strategies, ensuring the timely and effective deployment of NEs. Despite challenges such as high initial investment and limited data availability, the integration of AI in NEs production holds considerable promise for cost‐effective, scalable, and sustainable BC strategies. This review explores the intersection of AI and BC, highlighting current applications, key challenges, and future opportunities in AI‐enhanced BC and NEs mass production. It synthesizes recent advancements and identifies research gaps, providing a comprehensive overview of AI's evolving impact on crop protection. By optimizing NE production and reducing dependence on chemical inputs, AI contributes to improved biodiversity, alignment with global sustainability goals, and a more resilient agricultural future. These insights are valuable for researchers, practitioners, and policymakers working toward sustainable and inclusive pest management solutions. © 2025 The Author(s). *Pest Management Science* published by John Wiley & Sons Ltd on behalf of Society of Chemical Industry.

## Graphical abstract

Schematic overview of the graphical abstract illustrating an AI‐integrated framework for mass rearing, quality optimization, and precision release of NEs for sustainable pest management. The left section depicts *Trichogramma* and *Chrysoperla* being reared in automated chambers equipped with IoT‐enabled sensors for monitoring light, temperature, and humidity, alongside robotic arms and computer vision systems for efficient handling. The center section highlights AI‐driven data dashboards that track real‐time parameters such as growth, survival, and reproduction, incorporating ML‐algorithms, genetic markers, and automated alerts to ensure quality control. The right section shows precision insect release using drones guided by satellite‐based pest hotspot maps, emphasizing reduced chemical usage with a ‘no pesticide’ icon and promoting eco‐efficiency and sustainability designed using Biorender (www.biorender.com).

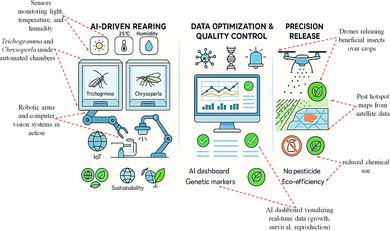

## 1 INTRODUCTION

The modern agricultural landscape faces growing challenges from pest infestations threatening crop yields plus global food security. Pest control methods that are customary, mainly because of their chemical pesticide use, have led to issues in both the environment and health that are quite serious, including pesticide resistance, biodiversity loss, and contamination of the food supplies.^1^ In response, there is a renewed interest in sustainable and environmentally friendly alternatives, particularly **BC** a pest management strategy that utilizes NEs, such as predators and parasitoids, to suppress pest populations.^2^, ^3^ Interestingly, Future Market Insights^4^ indicates that the ‘Agri Natural Enemy Pest Control Market’ is expected to grow substantially from 2025 to 2035, influenced by the rising demand for sustainable farming practices, heightened concerns regarding chemical pesticide residues, and progress in biological pest control technologies. The market is projected to grow from $19.2 billion in 2025 to $32.8 billion by 2035, indicating a compound annual growth rate (CAGR) of 5.8% over the forecast period. Key factors driving this growth are the global transition to organic farming, stringent regulations on synthetic pesticides, and an increasing awareness of integrated pest management (IPM) strategies. The pursuit of eco‐friendly alternatives to chemical pesticides has led to a rising demand for raptorial insects, parasitoids, microbial products, and beneficial organisms among growers. In addition, the adoption of perfection farming and AI‐based pest monitoring systems is enhancing the effectiveness of NEs‐based pest control results. The growing vacuity of customized BC results, bettered mass‐parenting ways, and distribution networks is further accelerating market expansion. Today's consumers are also willing to spend more on chemical‐free or organic foods.^4^ Additionally, governments and regulatory bodies worldwide are promoting biological pest control methods to reduce reliance on chemical pesticides, further supporting market expansion. For instance, the European Union (EU)'s ‘Green Deal’ (https://commission.europa.eu/strategy-and-policy/priorities-2019-2024/european-green-deal_en) and China's Kunming‐Montreal Global Biodiversity Framework (https://www.unep.org/resources/kunming‐montreal‐global‐biodiversity‐framework) aim to enhance sustainability, with the EU targeting a 50% reduction in pesticide use by 2030, and China pledging to restore 30% of its ecosystems.^5^

BC involves the use of NEs to manage pest populations.^6^, ^7^ These organisms can be mass‐produced and released into agricultural environments to control pests. Examples include *Harmonia* and *Coccinella* ladybirds, *Chrysoperla* lacewings, *Phytoseiulus* predatory mites, and parasitic wasps such as *Trichogramma*, *Cotesia*, and *Encarsia*. BC reduces dependence on chemical pesticides, promotes ecological balance, and supports global sustainable agriculture goals.^8^ However, large‐scale adoption of BC faces challenges, primarily related to the mass production and consistent quality of NEs. The production of species such as *Trichogramma spp*. is intricate and laborious, necessitating substantial optimization for cost‐effectiveness and operational efficiency. The production of one million *Trichogramma* wasps may cost between €20 and €40, depending on labor, material costs, and technology utilized.^9^ The procedure necessitates strict environmental regulations, consistent quality assessments, and skilled personnel, which complicates scaling. Manual handling introduces variability in NEs quality and extends manufacturing duration; AI offers effective solutions to these issues. Automation powered by AI and real‐time monitoring can decrease labor costs by 30–50% while improving consistency.^10^ This enhances profitability and strengthens the overall commercial viability of BC by enabling large‐scale, standardized NEs production.

In recent years, AI technology has developed into a significant tool in agriculture, offering intelligent solutions for production, monitoring, and decision‐making.^11^ Technologies such as machine learning (ML), computer vision, the Internet of Things (IoT), and robotics enable precise identification of pests, diseases, and weeds, thereby promoting environmentally sustainable crop protection strategies.^12^, ^13^, ^14^ AI has the potential to transform NEs mass production by automating labor‐intensive tasks, optimizing raising conditions, improving quality control, and enabling precision release systems.^15^ For example, Bjerge *et al*.^16^ presented a computer vision system was developed using YOLOv3 on an NVIDIA Jetson Nano to identify and categorize insects, achieving a mean average precision (mAP) of 87% across eight species. The system achieved real‐time tracking with an accuracy of 89%, processing images at a rate of 0.5 frames per second over a period of 98 days, and successfully identifying 2994 legitimate insect tracks. This provided insights into developmental stages, health, and behavior, while automating aspects of insect cultivation and quality assurance. Likewise, Kapetas *et al*.^17^ developed YOLOv10 deep learning model which was utilized to detect black aphids on sticky traps in cucumber greenhouses, achieving a mAP50 of 89.1%, precision of 84.8%, and recall of 87.7% at a resolution of 1600 × 1600. The mobile application enabled real‐time monitoring and early detection, while an integrated ARIMAX model predicted aphid populations with an average variance of only 8.61 insects per day. These examples show how AI can help maintain consistent production quality, quick detection of problems, and support fast decision‐making in mass rearing systems for NEs. ML algorithms are increasingly utilized in insect rearing to improve critical environmental factors such as temperature, humidity, photoperiod, and dietary composition, thereby enhancing the survival, development, and fecundity of new entities. Hurali *et al*.^18^ documented the implementation of AI‐driven temperature control systems that incorporate real‐time sensors and automated fertilizer delivery systems for *Trichogramma spp*. and *Phytoseiulus persimilis*. The application of random forest and decision tree models enabled dynamic adjustments to rearing conditions, resulting in a mortality reduction of up to 40% and an improvement in emergence rates exceeding 25% compared to traditional methods. Additionally, ML‐enhanced feeding systems have optimized protein‐carbohydrate ratios in synthetic diets, significantly enhancing fertility and larval survival in populations of *C. carnea* and *Aphidius colemani*. Intelligent systems reduce labor, minimize errors, and improve scalability through adaptive learning. Predictive models driven by AI and adaptive feedback systems can synchronize neurotoxin release with pest population dynamics, thereby enhancing efficacy and reducing waste.^3^, ^19^ Liang *et al*.^19^ developed models that ascertain optimal release quantities informed by pesticide resistance and cumulative NEs mortality, thereby facilitating effective pest management with minimal resource expenditure. The implementation of these measures could lead to a reduction in costs by as much as 30%, equating to $20–30 per hectare, depending on the specific crop type and geographic location. These systems often incorporate remote sensing data, real‐time monitoring instruments, and genomic information of pests and NEs to enable informed decision‐making. These advancements alleviate considerable limitations of traditional mass‐rearing techniques and improve the economic and practical viability of BC.

Nevertheless in spite of these promising developments, challenges remain to effectively leverage the capabilities of AI in BC, and it is crucial to address the constraints posed by limited datasets, significant upfront costs, the requirement for specialized technical expertise, and concerns related to data security.^20^ This review explores the capabilities of AI technologies in optimizing the efficient mass production of nanostructured entities for biocompatibility applications. The discussion highlights the benefits, challenges, and prospective developments of AI‐driven innovations in enhancing production efficiency, ensuring consistency, and advancing sustainable agricultural practices.

## 2 THE NEED FOR EFFICIENT MASS PRODUCTION OF NEs

**Figure 1:**
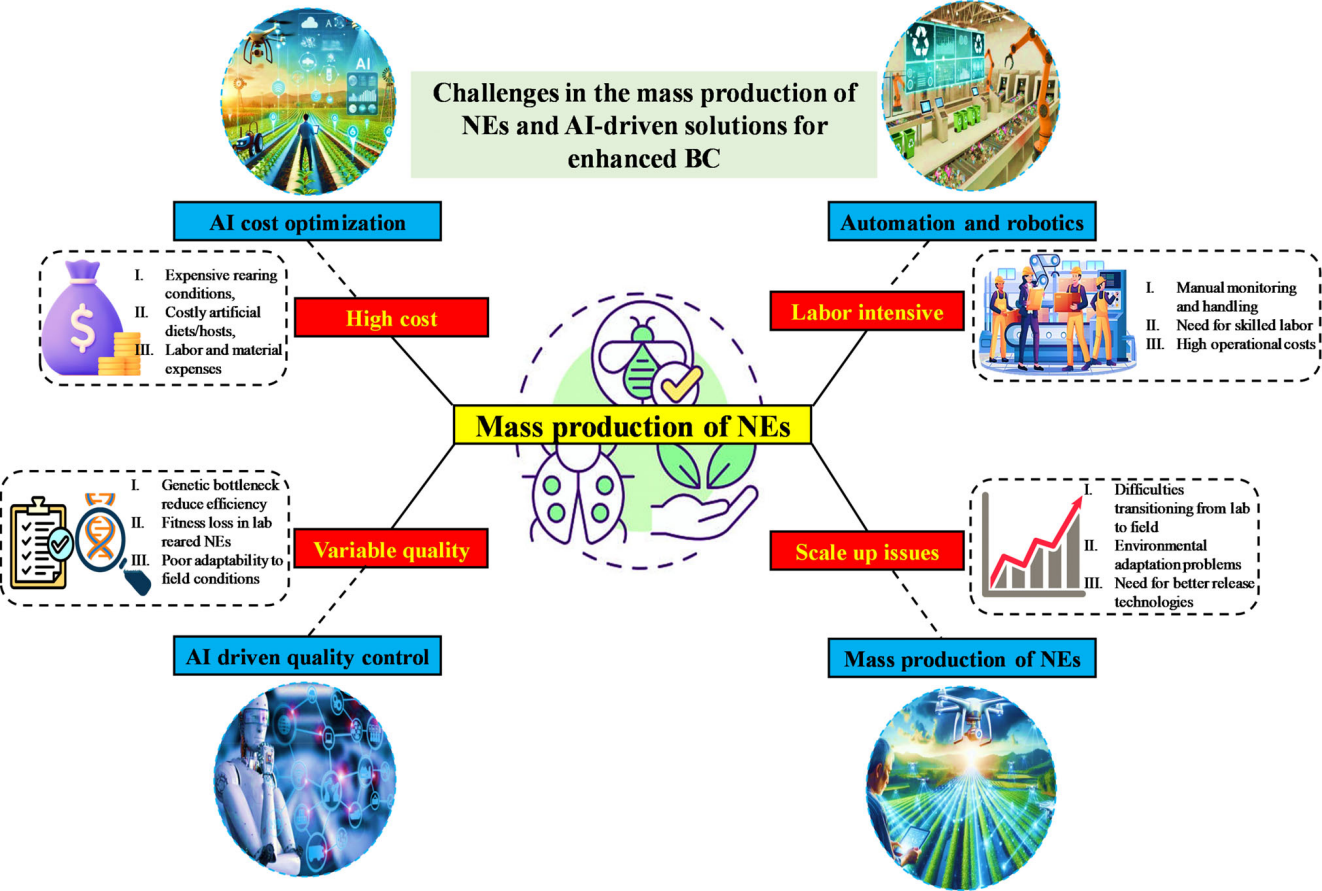
Schematic overview of the major challenges in the mass production of natural enemies for biological control, including high costs, labor‐intensive processes, variable quality, and scale‐up issues, also highlighting potential AI‐driven solutions, such as cost optimization algorithms, automated monitoring systems, data‐driven quality control, and predictive modeling, that can address these challenges to enhance modern agricultural practices designed using Biorender.com.

The mass production of NEs, including parasitoids and predators, is essential for effective BC. The introduction/augmentative release of natural predators or parasitoids into the field is frequently one of the most successful methods for pest control.^2^ Nevertheless, the mass manufacture of these NEs encounters several technical, economic, and biological obstacles that impede their extensive use (Fig. 1). This chapter rigorously analyzes these problems, offering examples and insights from contemporary research.

### 2.1 High costs associated with mass production

Producing substantial quantities of NEs can be costly, especially when ensuring ideal growth conditions and supplying appropriate food sources. Significant cost determinants encompass the necessity for regulated environmental circumstances, nutrient‐dense artificial feeds or natural hosts, infrastructure, and proficient labor. The mass raising of *Trichogramma spp*., a prevalent egg parasitoid worldwide, frequently relies on high‐quality host eggs from *Ephestia kuehniella* or *Corcyra cephalonica*.^21^, ^22^ The production of these artificial hosts can constitute up to 60% of overall operational expenses, with expenditures varying from $60 to $120 (€55–€110) per million host eggs, contingent upon the procedures and resources employed. Likewise, *C. carnea*, a generalist predator, necessitates protein‐dense diets for larval development, which are expensive to produce and scale. Artificial diets can range from $80 to $150 (€74–€138) per kilogram, which restricts their widespread use despite continuous research into economical alternatives. Recent advancements in conservation BC have investigated artificial meals for the cultivation of parasitoids such as *Trichogramma dendrolimi* to diminish dependence on natural hosts.^23^, ^24^ Semi‐artificial diets have potential; yet, issues including diminished fecundity and fitness over generations impede long‐term economic sustainability. Artificial feeds are being optimized to reduce expenses and standardize cultivation practices. The integration of AI further improves these initiatives by optimizing nutrient content, automating quality monitoring, and forecasting generational reductions.^18^ AI‐driven systems may regulate micro‐environmental variables, detect abnormalities in real time, and dynamically change feeding regimens, thereby enhancing cost‐efficiency and nutrient efficiency quality. The role of AI transcends laboratory settings. Gurr *et al*.^25^ employed AI techniques to examine the influence of nectar and pollen feeding on NEs efficacy, revealing that dicotyledonous plants providing both resources enhance BC results. Such insights facilitate the development of BC strategies that are both scalable and sustainable. Consequently, although reconciling cost reduction with NEs efficacy presents a problem, AI possesses the capability to enhance release timing, minimize redundant applications, and augment operational efficiency potentially decreasing costs by 30–50%, contingent upon the crop system and release strategy.

### 2.2 Labor‐intensive processes

The mass manufacturing of NEs is labor‐intensive, requiring manual oversight, cultivation, management, and packaging. This not only escalates expenses but also causes variability. Rearing *A. colemani* necessitates continuous observation of host aphid colonies, collection of mummified aphids, and meticulous regulation of emergence circumstances.^26^ Similarly, cultivating predatory mites such as *P. persimilis* necessitates the management of prey mite colonies, hence increasing labor complexity.^27^ Despite advancements in automated counting and sorting equipment (*e.g*., for *Trichogramma spp*.; Basana Gowda *et al*.^22^), issues persist regarding scalability, precision, and cost‐effectiveness. Artificial intelligence provides promising solutions *via* computer vision for the identification of host‐parasitoid relationships and machine learning techniques for the optimization of rearing settings. These technologies can diminish labor intensity and enhance consistency, efficiency, and real‐time decision‐making.

### 2.3 Variable quality

Ensuring uniform quality in mass‐produced NEs continues to be a significant difficulty. Genetic drift, inbreeding, and suboptimal rearing conditions can diminish field performance.^2^ *Cotesia plutellae*, employed against *Plutella xylostella*, has exhibited diminished parasitism efficiency and host‐seeking behavior during multiple laboratory generations as a result of inbreeding depression.^28^ Koppert Biological Systems and similar companies currently employ AI‐driven quality control solutions. AI‐driven image recognition systems evaluate characteristics such as wing symmetry, size, and flight behavior in *Trichogramma brassicae*, employing convolutional neural networks (CNNs) to detect anomalies and behavioral discrepancies. The immediate elimination of substandard individuals enhances parasitism rates and adaptation in the field. Moreover, AI‐assisted environmental control systems dynamically regulate temperature, humidity, and photoperiod utilizing sensor data to minimize developmental variability. For species such as *Orius insidiosus*, the incorporation of genetic management strategies such as the periodic introgression of wild genes^29^ combined with AI‐driven quality control guarantees resilient and efficient natural enemy (NEs) populations.

### 2.4 Scale‐up issues

Transitioning from laboratory to commercial production presents technological and logistical obstacles. Laboratory circumstances seldom mimic field environments, resulting in diminished survival and effectiveness after release. Lab‐reared *Cryptolaemus montrouzieri* frequently struggles to acclimate in orchards due to temperature variations and insufficient prey.^30^ Entomopathogenic fungi (EPF), such as *Beauveria bassiana*, encounter challenges in formulation and application owing to their susceptibility to ultraviolet light and humidity.^31^, ^32^ Research is being conducted on solutions like microencapsulation to enhance spore stability and drone‐based dispersal.^33^ Scaling the production of *Trichogramma spp*. poses challenges: generating one million wasps requires 10–12 days and incurs significant expenses related to host egg production, manpower, and quality assurance.^34^, ^35^ AI‐assisted sorting, enhanced rearing techniques, and environmental automation are necessary to tackle these issues. These tactics diminish time, effort, and expenses while guaranteeing the quality and field efficacy of parasitoids.

## 3 AI IN OPTIMIZING THE REARING PROCESS FOR NES

**Figure 2:**
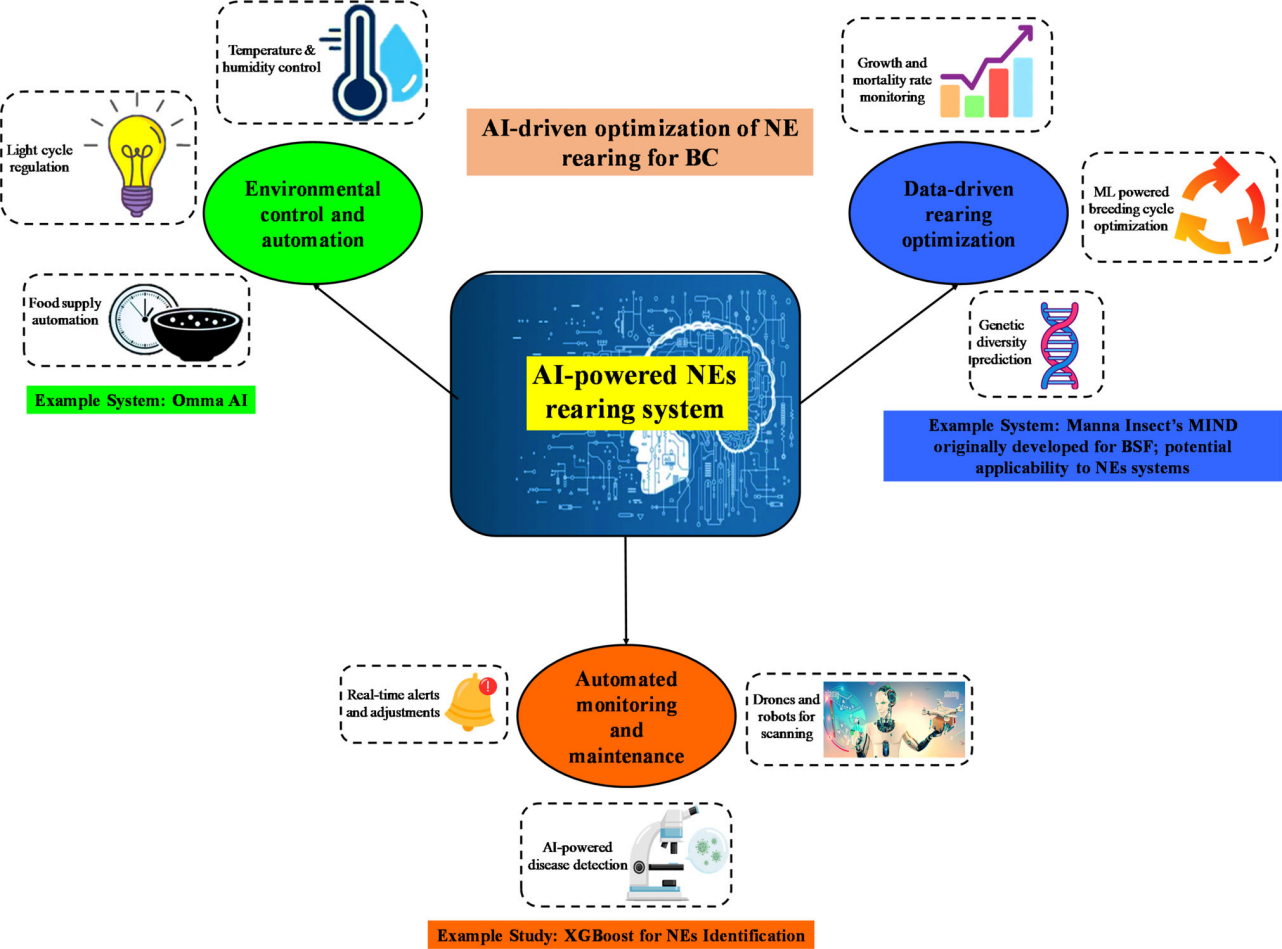
The role of AI in optimizing the mass rearing of NEs for BC. The AI‐powered system integrates three key components: (1) environmental control and automation, (2) data‐driven rearing optimization (3) automated monitoring and maintenance. Environmental control and automation: AI‐driven sensors regulate key environmental factors such as temperature, humidity, CO_2_ levels, and light cycles to optimize NEs growth and reproduction. Automated feeding systems ensure consistent food supply, while AI algorithms dynamically adjust conditions to enhance survival and reproductive rates. An example system, Manna Insect's MIND, originally designed for BSF farming, demonstrates the potential for adapting AI‐regulated climate control to NEs rearing. While not a direct application to BC, it illustrates the scalability of such platforms for insect mass production. Data‐driven rearing optimization: ML models analyze large datasets on growth rates, mortality, and reproductive success to optimize rearing conditions. AI predicts peak reproductive phases, adjusts feeding schedules, and optimizes genetic diversity for robust NEs populations. The Omma AI modular system enables on‐farm rearing of beneficial insects for continuous and preventative pest control. Automated monitoring and maintenance: AI‐powered vision technologies, drones, and robotic systems continuously monitor NEs health, detect diseases, and identify rearing inefficiencies. Computer vision algorithms analyze real‐time behavioral and morphological data, while automated alerts help prevent production bottlenecks. XGBoost‐based AI systems have achieved high accuracy in identifying predatory mites for IPM, demonstrating AI's potential in NEs species identification and quality control. By integrating AI into NEs mass production, this approach enhances efficiency, reduces costs, and supports sustainable pest management in modern agriculture. Designed using Biorender.com.

The bulk production of NEs for BC in contemporary agriculture requires accurate and effective rearing methods. A key component is rearing NEs in controlled environments that simulate natural conditions. AI provides transformative solutions to optimize these processes,^36^ improving the effectiveness and scalability of BC approaches (Fig. 2). AI based technologies can enhance the raising process in various ways, as outlined below.

### 3.1 Environmental control and automation

Ensuring adequate environmental conditions is essential for the effective rearing of NEs. AI‐driven environmental control systems may oversee and adjust parameters within rearing facilities, such as temperature, humidity, light cycles, and CO_2_ concentrations. ML‐ algorithms evaluate real‐time data from integrated sensors to guarantee optimal growth and reproduction conditions. These systems may autonomously modify settings in reaction to environmental variations, ensuring a consistent environment for large‐scale production. AI systems are revolutionizing the cultivation of *Trichogramma* wasps commonly utilized egg parasitoids in BC by scrutinizing extensive statistics on temperature, humidity, host egg quality, and photoperiod to optimize conditions for parasitism and reproductive efficacy. These adaptive systems dynamically modify environmental parameters to optimize survival and reproductive success across generations, minimizing trial‐and‐error and operational expenses. Similarly, in the mass production of predators such as *Chrysoperla spp*. (green lacewings), AI plays a key role in regulating breeding cycles and managing nutritional regimes (*e.g*., protein‐rich artificial diets or prey availability). Precise control over variables such as larval density, humidity, and light cycles enhances egg production and larval development, ensuring consistent predator quality and better field performance. While originally developed for BSF production, Manna Insect's MIND (https://www.mannainsect.com/manna-mind-bsf-farming-technology/) demonstrates the potential adaptability of AI‐powered environmental control platforms to NEs rearing. Such cross‐applications underline the technological feasibility of scaling AI systems for biological control applications. This system precisely regulates climate conditions and lighting across insect life stages and can be adapted to NEs, enabling reproducible and scalable insect production across various rearing facilities. Although BSF is not used as NEs, the underlying rearing technologies are structurally similar and provide a technical reference point for NEs production systems.

### 3.2 Data‐driven rearing optimization

AI‐based systems collect vast amounts of data from the rearing process, including metrics such as growth, mortality, and reproductive success. Using ML models, AI can uncover patterns and relationships, enabling the development of predictive models to optimize rearing protocols. This may involve adjusting feeding schedules, modifying habitat configurations, or selecting breeding pairs based on performance. For example, AI can predict the peak reproductive phase of NEs, helping producers’ time of harvesting and field release for maximum pest control. ML‐algorithms can facilitate the control of genetic variation in mass‐reared populations, hence assuring resilience and adaptation. The latest innovation *i.e*. Omma has just introduced an AI‐driven modular system that automates the on‐farm raising of beneficial insects (https://www.antler.co/blog/why-we-invested-in-omma-ai-powered-technology-which-enables-growers-to-rear-beneficial-insects-on-farms-for-natural-pest-management/). This method allows cultivators to breed generalist insects on‐site, facilitating continuous and proactive pest management through automated pest detection.

### 3.3 Automation of monitoring and maintenance

AI‐driven robotics and computer vision technologies can automate the surveillance and upkeep of NEs in raising facilities. These systems perpetually evaluate organism health and behavior, identifying problems like disease outbreaks or physical stressors that could affect quality and yield. Drones and robots outfitted with AI may survey raising units and notify operators of any issues, so minimizing labor and human error. For instance, drone‐based AI systems can identify behavioral alterations such as heightened mortality or diminished reproduction and initiate quick remedies. A significant work by Liao *et al*.^37^ utilized extreme gradient boosting (XGBoost) to automate the identification of *Neoseiulus barkeri* and 35 associated *Phytoseiidae* mite species. The model attained 100% accuracy on the complete dataset and 99% during cross‐validation, utilizing 22 morphometric features from 512 female specimens, with no false positives or negatives recorded. Feature importance analysis revealed seta j4 as the most critical attribute, however ICE plots demonstrated the non‐linear influences of features such as J5 and j3. This method facilitates precise and interpretable identification, allowing knowledge transfer from taxonomists to non‐specialists, and establishes a foundation for scalable, AI‐driven monitoring of biological control agents (BCAs). Moreover, AI technologies facilitate the monitoring of food consumption and growth rates, providing early alerts regarding inefficiencies or production constraints. Avila *et al*.^38^ created a ‘smart bin’ system that incorporates sensors, thermal imaging, and computer vision to monitor BSF larvae growth and environmental conditions, hence minimizing manual intervention. Hurali *et al*.^18^ examined the integration of AI and robotics advances in mass‐rearing facilities, which enhances environmental control, reduces costs, and improves quality assurance. Furthermore, while outside the primary scope of NEs‐based BC, advancements in AI‐assisted pollinator rearing reinforce the broader applicability of AI to insect‐based agricultural services. AI‐supported controlled environments provide reliable, high‐quality pollination services, enhancing both health and field performance. These achievements illustrate the revolutionary capacity of AI and automation in the large‐scale production of beneficial insects, including both NEs for pest control and pollinators, thus enhancing sustainable and efficient agriculture operations.

## 4 AI IN OPTIMIZING RELEASE STRATEGIES

**Figure 3:**
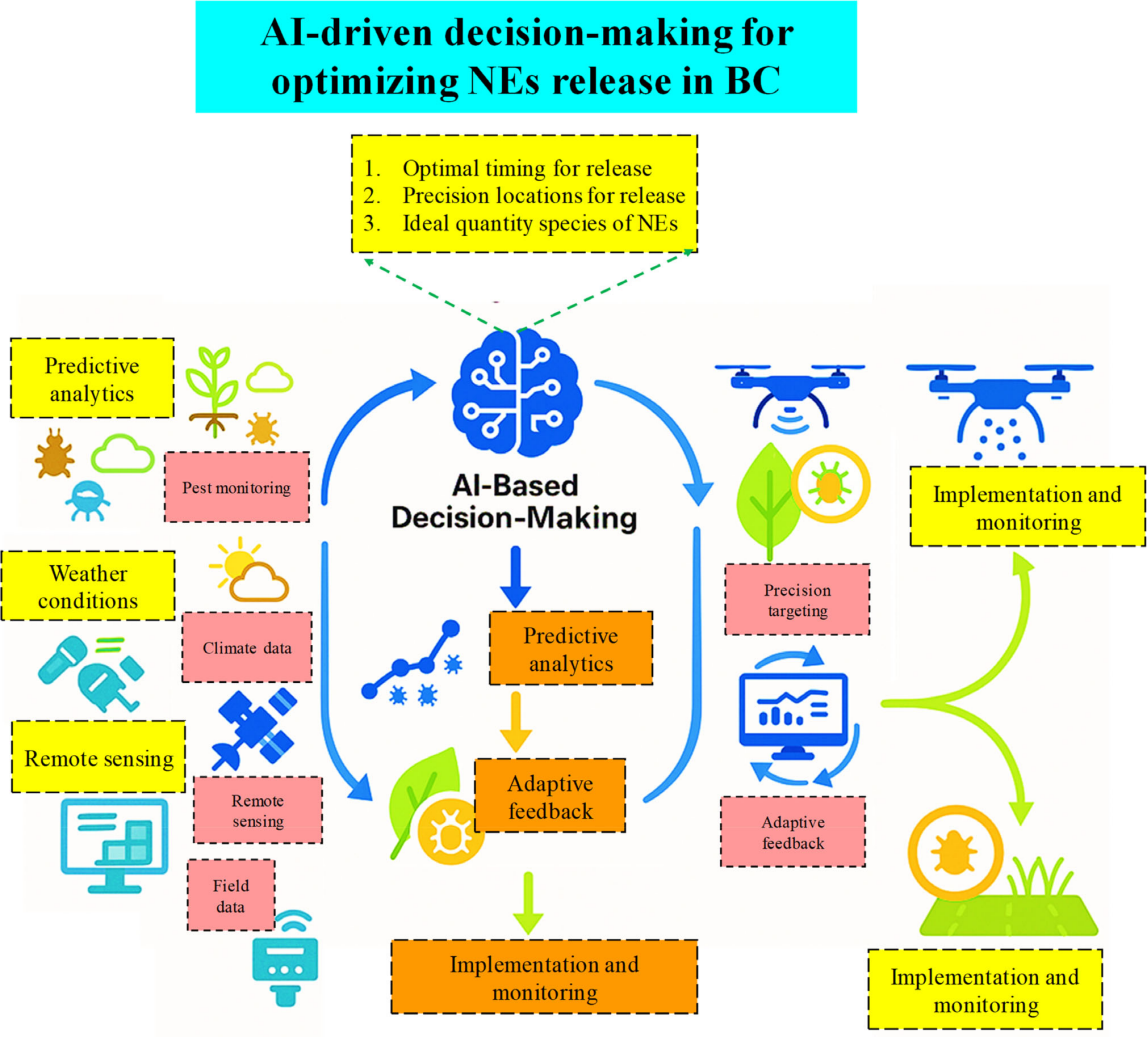
The schematic illustrates the role of AI‐driven decision‐making in optimizing the mass release of NEs for biological pest control. AI integrates multiple data sources, including predictive analytics, weather conditions, remote sensing, and field data, to determine the optimal timing, precise release locations, and ideal quantity and species of NEs. The AI system processes this information to generate actionable insights, which are then implemented and monitored using advanced technologies such as drones and field‐based monitoring systems. This approach enhances the efficiency and effectiveness of BC strategies by ensuring precise and adaptive pest management interventions. Designed using Biorender.com.

The efficacy of BC initiatives predominantly relies on the successful introduction of mass‐produced NEs into agroecosystems. Critical factors namely timing, location, and release quantity significantly affect the effectiveness of NEs in controlling insect populations. Historically, release tactics have depended on empirical knowledge, regular field scouting, and generalized pest development models. Integrating AI presents transformative possibilities, facilitating data‐driven, adaptive, and precision‐based release tactics that optimize BC results. Figure 3 illustrates a schematic representation of AI‐facilitated decision‐making for the optimization of NEs release in BC. AI technologies can augment the accuracy and efficiency of these releases in multiple significant ways:

### 4.1 Predictive analytics for pest populations

AI‐driven predictive algorithms can evaluate historical and real‐time data regarding pest behavior, meteorological conditions, and crop health to ascertain the ideal timing for NEs releases.^39^ ML‐algorithms can analyze data from sensors, satellite photography, and terrestrial observations to forecast when pest populations will attain critical levels and when NEs are expected to be most efficacious.^40^ Predictive models that use multi‐source data, including weather trends, remote sensing imaging, and field surveillance, have been utilized to anticipate aphid outbreaks in wheat. Luo *et al*.^41^ utilized a Backpropagation Neural Network (BPNN) to forecast wheat aphid occurrence, whereas Skendžić *et al*.^42^ applied Support Vector Machines (SVM) and Random Forest (RF) algorithms in conjunction with hyperspectral canopy reflectance data to categorize aphid‐infested wheat. These models provide the prompt introduction of aphid‐specific parasitoids such as *A. colemani*, optimizing effectiveness as pest populations near economic thresholds. Likewise, for *Bemisia tabaci*, AI‐models such as a bagging ensemble artificial neural network have attained significant accuracy (R2 = 85.57%) in predicting infestation peaks by utilizing data from primary plant nodes where whiteflies congregate.^43^ Although this model does not yet differentiate parasitized from non‐parasitized individuals, integration with AI‐powered image analysis could fill this gap. For example, companies like Biobest Group are already using AI to detect whiteflies; extending this to assess parasitism rates could greatly enhance BC precision. By predicting pest hotspots and temporal patterns, AI diminishes dependence on prophylactic releases and pesticides, fostering a more targeted, sustainable, and efficient BC strategy.

### 4.2 Precision release technologies

**Figure 4:**
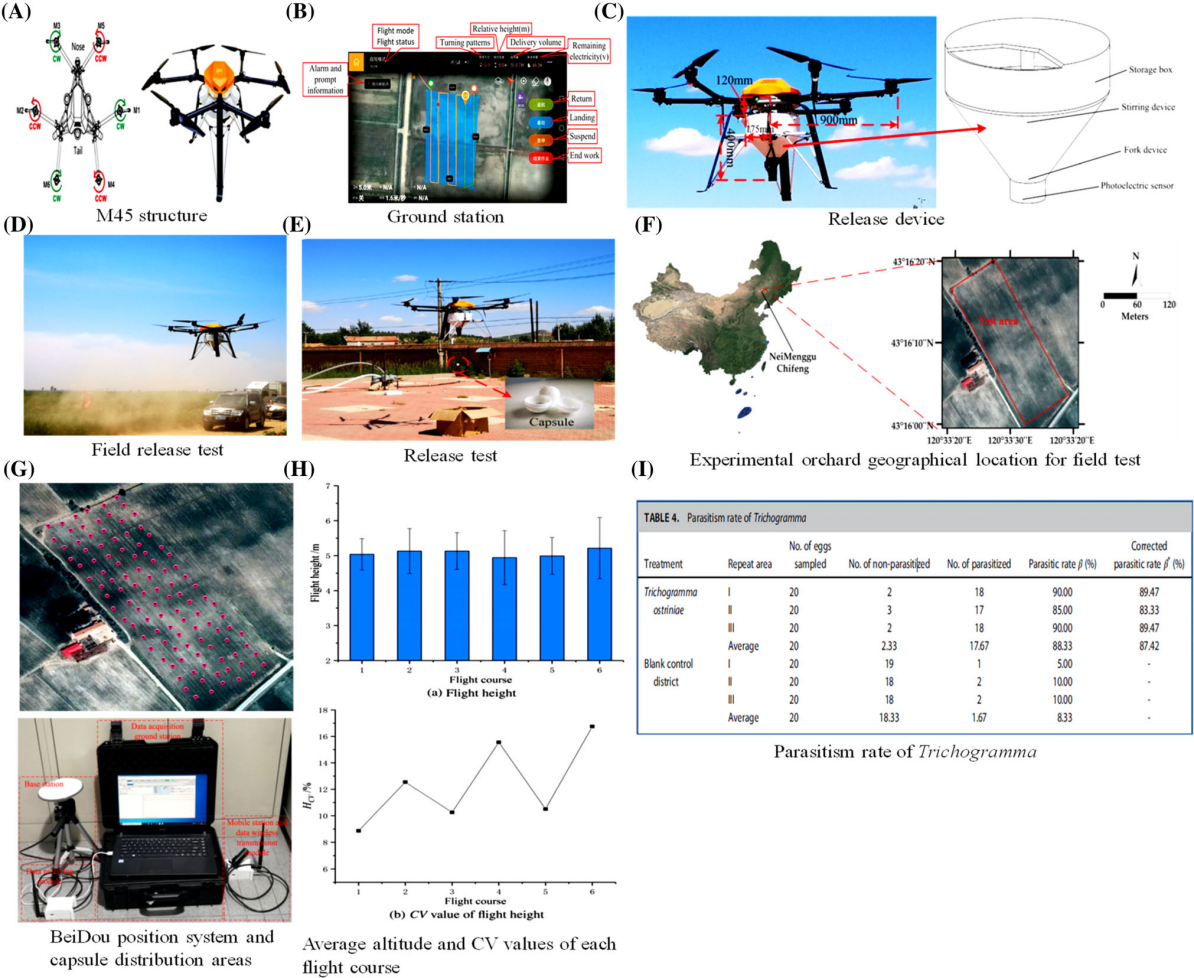
The intelligent UAV‐based delivery system for BC with *Trichogramma ostriniae* capsules. (A) M45 electric multirotor UAV structural schematic together with motor arrangement. (B) Ground control station interface showing aircraft status, flight path, release rate, battery level. (C) Key components including the storage box, stirring mechanism, fork, and photoelectric sensor are shown on a UAV‐mounted natural enemy capsule delivery system. (D‐E) Field release activities and aerial capsule design. (F) Mapped test area and geographic location of the Chifeng, Inner Mongolia test site. (G) Actual field distribution places of freed capsules and visual route map. (H—I) Flight height over six UAV flight paths is shown in a bar chart; height stability is shown by coefficient of variance (CV) displaying notably better parasitism in treatment plots, table displaying parasitism rate and corrected parasitism rate of *T. ostriniae* in released *vs*. control regions. Supporting its efficiency for precision biological control, the integrated UAV system shows good flying accuracy, steady altitude, uniform capsule distribution, and a corrected parasitism rate of 87.42%. Modified from Zhan *et al*.^49^ and Martel *et al*.^44^

AI can be integrated with precision release technologies, like drones or unmanned aerial systems (UAS), to effectively distribute NEs throughout extensive or intricate agricultural terrains. These technologies provide the precise allocation of NEs to areas afflicted with pests, enhancing effectiveness and minimizing resource wastage. Recent studies illustrate the feasibility of drone‐based BC. Martel *et al*.^44^ documented the effective aerial release of *Trichogramma* parasitoids to manage *Ostrinia nubilalis* in maize and *Choristoneura fumiferana* in coniferous woods, attaining elevated parasitism rates and diminishing manual labor requirements. Likewise, Song *et al*.^45^ created an agricultural drone system proficient in the uniform distribution of *Trichogramma* balls, incorporating AI‐driven geolocation and pest data integration to optimize coverage and timing. AI based algorithms can facilitate drone navigation and modify NEs amounts according to real‐time pest density information. Intelligent swarming algorithms provide the coordinated deployment of many drones, guaranteeing extensive coverage in diverse situations such as orchards or greenhouses.^46^ Additional innovation encompasses an AI‐assisted unmanned aerial vehicle (UAV) system for the deployment of *Harmonia axyridis* in cucumber greenhouses. This system employed deep learning‐based image analysis to detect aphid hotspots and modify release rates accordingly, leading to substantial decreases in both aphid populations and pesticide application.^47^, ^48^ Another system developed by Zhan *et al*.^49^ (Fig. 4) used an M45 multirotor drone to release *Trichogramma ostriniae* in cornfields, achieving 99.33% effective coverage and 83.70% pest control. These autonomous systems offer a cost‐effective and precise alternative to manual BC methods.

### 4.3 Adaptive feedback systems

AI assists BC not only during the planning phase but also by facilitating adaptive feedback mechanisms for ongoing strategy enhancement. These systems amalgamate continuous pest surveillance, environmental data, and NEs performance indicators to adaptively modify release strategies. Reinforcement learning algorithms can evaluate field data in real time and adjust methods by altering release frequencies, amounts, or even changing NEs species when required.^50^ If pest control is inadequate, the system may suggest more releases or modifications depending on climatic conditions, resistance development, or ecological dynamics. A pilot study utilized an AI‐driven feedback loop that integrated pheromone trap data with climatic variables to optimize the release of *C. carnea* for thrips control in pepper crops. The system dynamically modified release patterns in reaction to pest reappearance, enhancing control results while minimizing NEs usage.^51^ By integrating real‐time feedback, AI systems may continuously refine BC strategies, ensuring resilience to field variability and enhancing long‐term pest management and ecological balance. In summary, AI is revolutionizing the integration of network components in business continuity plans. Predictive analytics improve release scheduling, precision technologies enable targeted applications, and adaptive feedback systems allow for dynamic, real‐time decision‐making. These advancements collectively improve the effectiveness, sustainability, and environmental accountability of pest management methods.

## 5 AI IN QUALITY CONTROL AND CONSISTENCY

**Figure 5:**
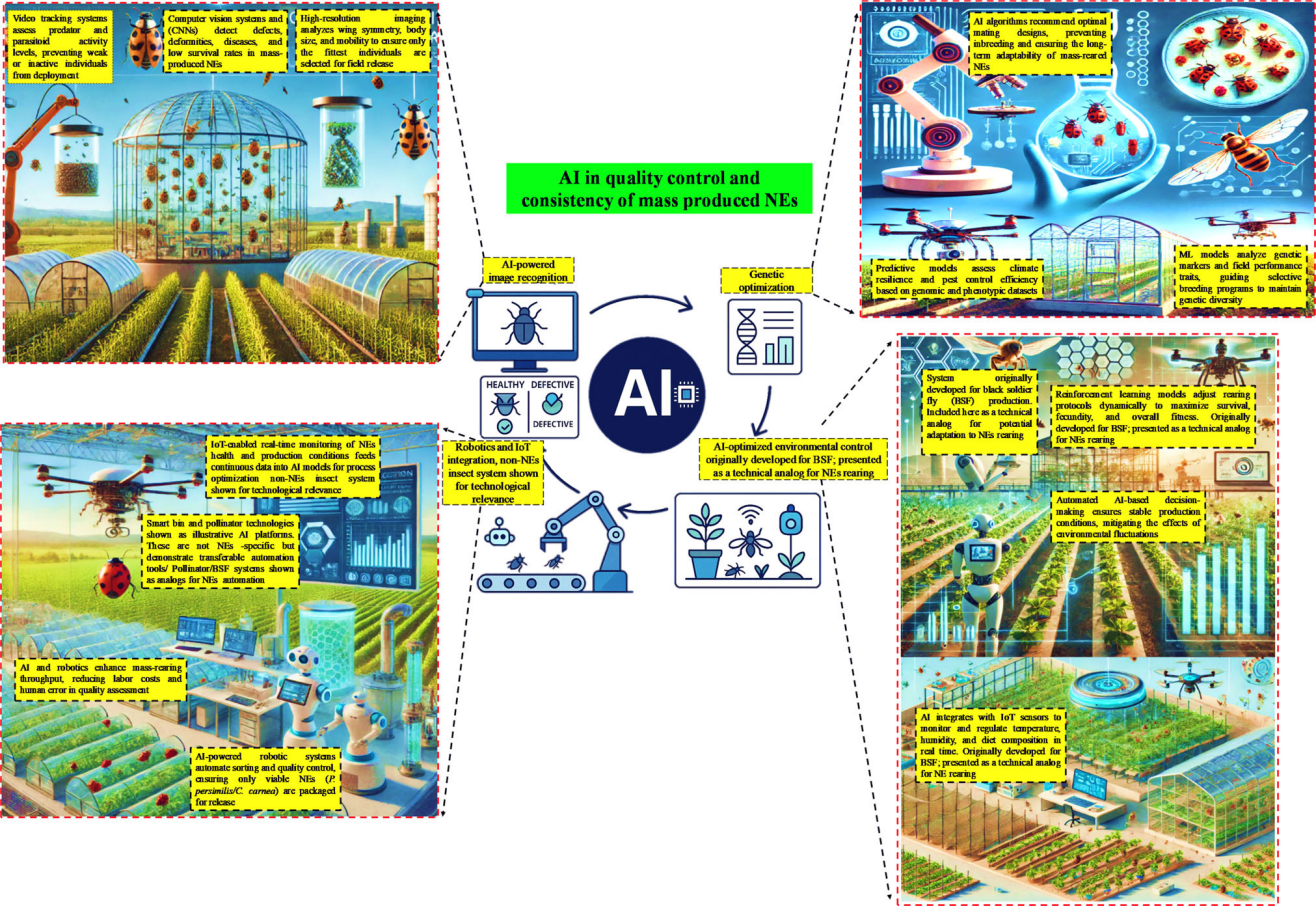
The schematic illustrates the in‐depth role of AI in quality control and consistency of mass‐produced NEs. Some elements shown (*e.g*., smart bin systems and pollinator platforms) are included as technological analogs. While they originate from non‐NEs insect systems like BSF or bumblebees, they demonstrate relevant AI applications that may be adapted for NEs‐based BC. This conceptual sketch shows how AI‐technologies transform mass production of NEs for BC in agriculture's consistency and quality control. Four fundamental AI‐driven domains AI‐powered image recognition for defect detection in NEs, genetic optimization to improve fitness and adaptability, AI‐powered environmental control for stable production, and robotics and IoT integration for automation and monitoring are highlighted in the central cycle diagram. Top Left Panel: To separate viable from faulty NEs, computer vision systems including CNNs detect physical defects, illnesses, and movement problems. Before field release, video tracking systems examine behavioral patterns including parasitism or predator–prey activities, therefore allowing early screening. AI algorithms provide ideal breeding plans, therefore guaranteeing the survival, fecundity, and adaptability of NEs over generations. By means of analysis of fitness, pest control capacity, and tolerance to climatic stress, ML‐models direct genetic selection and thereby preserve genetic diversity. Using genetic and phenotypic data, predictive models increase biocontrol performance and climate resilience. IoT sensors offer real‐time monitoring of NEs health, productivity, and environmental aspects, which feeds into AI models to improve processes. Bottom left panel: Robotic systems driven by AI sort and quality check to guarantee only fit NEs are packed for use. Some elements (*e.g*., BSF smart bins, pollinator platforms) are shown as technology analogs. While not NEs‐based systems, they demonstrate AI tools relevant to NEs production and quality control. Reducing human error and labor expenses, robotics increase throughput. By means of reinforcement learning algorithms, AI systems dynamically modify rearing settings to enhance survival and reproductive success. Platforms for automated decision‐making help to stabilize production settings and lower the effect of environmental changes. To guarantee best raising conditions, IoT‐enabled artificial intelligence constantly analyzes and controls variables including temperature, humidity, and diet composition. Designed using Biorender.com.

The effectiveness of BC programs is largely dependent on the extensive development of high‐quality NEs that exhibit optimal fitness^39^, ^52^ together with strong predatory or parasitic efficiency and resilience under field conditions. Maintaining constant quality in large‐scale production poses significant challenges due to biological diversity, labor‐intensive supervision, and fluctuating environmental conditions. AI, with its refined capabilities in automation, image recognition, and data‐driven decision‐making, provides unique ways to improve quality control and consistency in NEs mass manufacturing (Fig. 5). AI technologies may automate inspection and evaluation processes, ensuring that only the most viable and effective agents are selected for release, hence improving the overall efficacy of BC programs.

### 5.1 Image recognition for quality assessment

**Figure 6:**
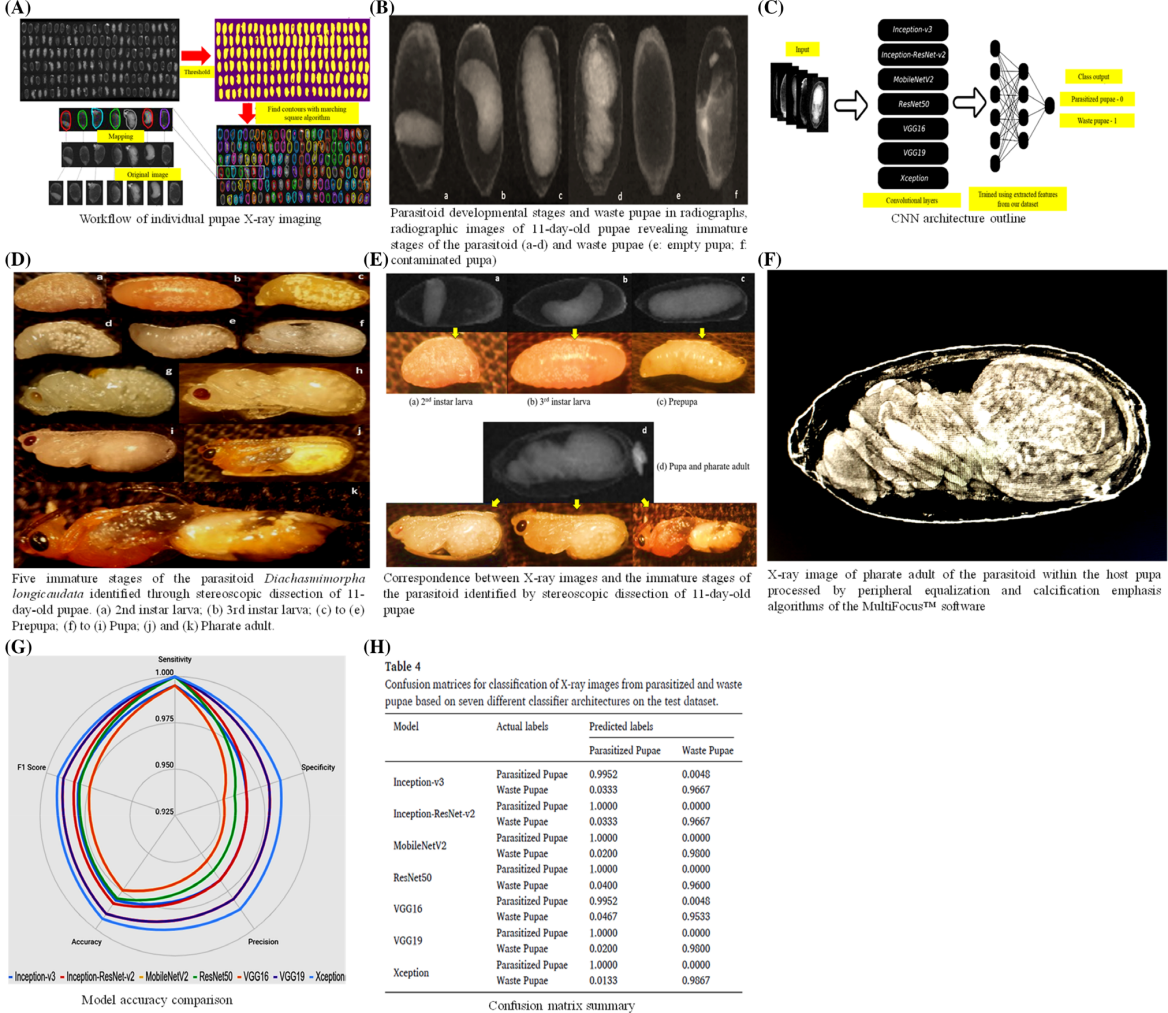
AI‐based classification of parasitized fruit fly pupae using CNNs and X‐ray imaging. (A) Workflow of individual pupae X‐ray imaging. (B) Parasitoid developmental stages and waste pupae in radiographs, radiographic images of 11‐day‐old pupae revealing immature stages of the parasitoid (a–d) and waste pupae (e: empty pupa; f: contaminated pupa). (C) CNN architecture outline. (D) Five immature stages of the parasitoid *Diachasmimorpha longicaudata* identified through stereoscopic dissection of 11‐day‐old pupae. (a) 2nd instar larva; (b) 3rd instar larva; (c) to (e) Prepupa; (f) to (i) Pupa; (j) and (k) Pharate adult. (E) Correspondence between X‐ray images and the immature stages of the parasitoid identified by stereoscopic dissection of 11‐day‐old pupae. (F) X‐ray image of pharate adult of the parasitoid within the host pupa processed by peripheral equalization and calcification emphasis algorithms of the MultiFocus™ software. (G) Model accuracy comparison. (H) Confusion matrix summary. Modified from Marinho *et al*.^53^

AI‐driven computer vision systems can autonomously inspect mass‐produced NEs, ensuring adherence to set quality standards. These systems can detect physical defects, signs of disease, or reduced vitality, all of which may affect the agent's effectiveness in the field. By analyzing attributes such as size, behavior, and activity levels, AI systems can assess the health and fitness of individuals, ensuring that only the most robust are selected for release. Models trained on comprehensive datasets may distinguish between healthy and suboptimal individuals based on morphological, behavioral, and physiological traits. CNNs, commonly utilized in plant disease detection, can be adapted for nutrient efficiency quality evaluations. These systems can identify deformities, discoloration, parasitic infections, and other factors that may compromise the efficacy of parasitoids, predators, or entomopathogenic agents (Fig. 6).^53^ Similarly, DL‐algorithms have been employed to assess the viability of *Trichogramma* species by analyzing wing symmetry and body size from high‐resolution images, achieving up to 95% accuracy in detecting individuals unfit for release due to developmental defects. Zhou *et al*.^54^ combined real‐time computer vision with robotic systems to assess the activity levels of *P. persimilis* during production, identifying batches with diminished mobility a vital sign of predator health. In addition to morphological evaluation, AI systems can examine behaviors including flying capability, host‐seeking behavior, and predatory efficiency through video tracking and machine learning models. These evaluations guarantee that only engaged, high‐performing individuals are permitted to proceed. The use of AI‐driven inspections mitigates human error, enhances throughput, and offers a consistent, objective methodology for quality evaluation, so overcoming the intrinsic limits of manual screening.

### 5.2 Data‐driven optimization of production protocols and genetic quality

AI can also enhance the genetic integrity of mass‐produced non‐engineered organisms. Repeated raising cycles frequently result in inbreeding, decreased genetic variety, and impaired field performance a well‐documented concern in BC projects.^55^ AI‐based tools and methods, including genetic algorithms and data analytics platforms, can evaluate population genetics to preserve genetic variety and resilience across future generations. Moreover, AI can discern genetic features associated with enhanced pest management efficacy, therefore informing breeding initiatives. For instance, ML methods can evaluate genetic and phenotypic datasets to forecast the fitness and field adaptation of diverse NEs strains. Khan *et al*.^56^ demonstrated the use of AI‐driven predictive models to correlate genetic markers with performance traits in *A. colemani*, hence supporting selective breeding strategies aimed at improving parasitism rates and climatic resilience. AI algorithms can provide optimal mating strategies or suggest the regular incorporation of wild strains to improve genetic diversity. This ensures the continued consistency and effectiveness of NEs populations. Moreover, AI can enhance environmental factors such as temperature, humidity, and nutritional composition by analyzing real‐time production data. Reinforcement learning models, which progress through trial and error, can enhance rearing conditions to facilitate survival, reproductive success, and host‐seeking behavior.^56^ In conclusion, the integration of AI into the quality control and production consistency of NEs presents revolutionary opportunities for BC programs. AI‐driven image recognition technologies deliver rapid, accurate, and consistent assessments of NEs quality, while data‐centric models ensure the maintenance of genetic integrity and the improvement of production processes. As AI technologies advance, particularly in conjunction with IoT sensors and robotics, they offer improved automation and precision. This will guarantee that only the most resilient, genetically varied, and behaviorally proficient NEs are utilized, hence optimizing the efficacy of pest management measures. Table 1 summarizes the role of AI technologies across various stages of NEs production and release, highlighting real‐world implementations and associated operational benefits.

**Table 1:** Summary of AI applications across key phases of mass production and deployment of natural enemies (NEs) in biological control (BC). This table highlights specific AI tools, their functional roles in NEs systems, the operational benefits they offer, and real‐world implementations or published examples

Production stage	AI technologies	Core functions	Operational benefits	Examples and use cases
Environmental control and automation	ML, IoT, Reinforcement learning	Real‐time adjustment of temperature, humidity, CO_2_, photoperiod; climate control across insect life stages	Improved NEs survival, fecundity, and fitness; reduced labor; lower resource input	Manna Insect's MIND for BSF; AI‐regulated rearing chambers for *Trichogramma spp*. and *Chrysoperla spp*.
Data‐driven optimization of rearing protocols	Predictive Modeling, Genetic algorithms, ANNs	Forecasting reproductive peaks; adjusting feeding density; selective breeding for genetic resilience	Consistent NEs quality; minimized inbreeding; enhanced reproductive success	Omma AI modular system for on‐farm rearing; AI‐genomic prediction in *Aphidius colemani* (Khan *et al*.^56^)
Monitoring and maintenance automation	Computer Vision (CV), Robotics, Drones, XGBoost	Continuous health and behavior surveillance; detection of mortality, stress, and disease; automated feeding and growth tracking	Early anomaly detection; reduced labor; greater batch‐level consistency	Liao *et al*.^37^: 99% accuracy in *Neoseiulus barkeri* ID; Avila *et al*.^38^: ‘smart bins’ for BSF
Quality control and assurance	CNNs, DL, Video Tracking Systems	Detection of deformities, wing asymmetry, and inactive individuals; assessment of mobility, parasitism, and flight behavior	Higher parasitism/ predation success; removal of poor performers; standardized QC	Zhou *et al*.^54^: visual QC for *Trichogramma* and *P. persimilis*; Marinho *et al*.^53^: X‐ray + CNN for *D. longicaudata* pupae
Release strategy optimization	Predictive Analytics, GIS, UAV Swarming Algorithms	Timing and location optimization; pest forecasting; automated drone deployment	Minimized pest resurgence; reduced pesticide need; scalable, efficient NEs deployment	Zhan *et al*.^49^: UAV release of *T. ostriniae*; Song *et al*.^45^: AI‐calibrated *Trichogramma* balls; UAV‐*Harmonia axyridis* deployment
Adaptive feedback and continuous learning	Reinforcement Learning, Real‐Time Sensor Analytics, Closed‐Loop Feedback Systems	Real‐time adjustment of release frequency/species; integration of NEs field performance and pest recurrence data	Improved ecological adaptability; real‐time decision‐making; reduced waste	Feedback loop‐based *C. carnea* release for thrips (Rodríguez and Coy‐Barrera^51^); AI‐linked pest monitoring platforms

## 6 CHALLENGES AND CURRENT GAPS

**Figure 7:**
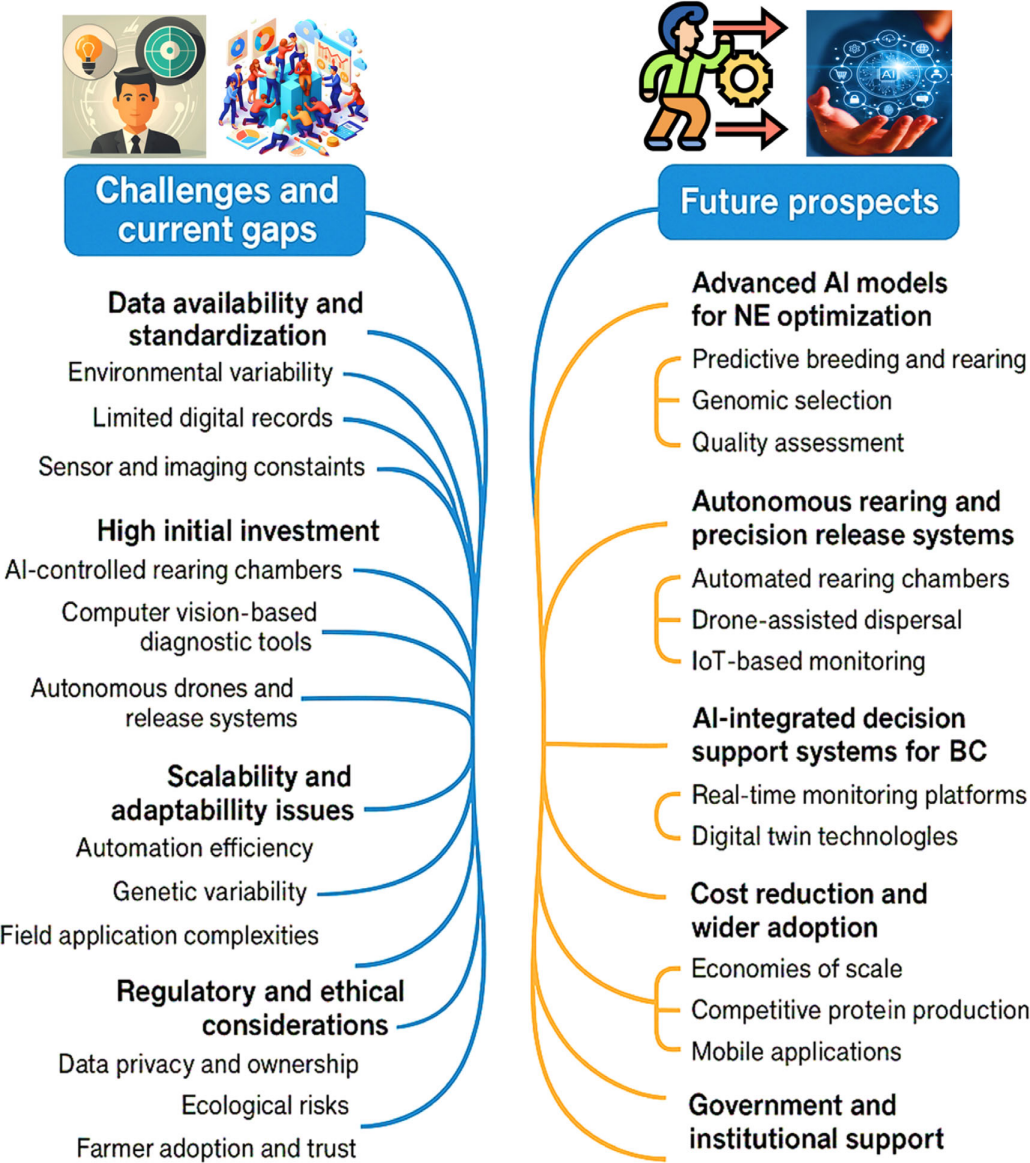
Prospective possible challenges, limitations ethical risks and future directions of AI in crop protection. Created with BioRender.com.

While the integration of AI into the mass production of NEs for BC holds significant promise, several technical, economic, and operational challenges must be addressed to enable large‐scale adoption and long‐term sustainability (Fig. 7).

### 6.1 Data availability and standardization

The performance of AI systems heavily depends on access to large, diverse, and high‐quality datasets. In the context of NEs mass production, AI models such as ML‐algorithms for lifecycle prediction or computer vision for health monitoring require comprehensive and well‐annotated training data. However, the agricultural sector faces several data‐related limitations:

- Environmental variability: AI systems struggle to generalize across fluctuating environmental conditions, such as temperature (ranging from 10 °C to 40 °C), humidity (30% to 95%), and varying photoperiods across agro‐ecological zones. These fluctuations introduce noise and inconsistencies in training datasets.
- Limited digital records: Many BC systems lack digitized historical records on NEs performance, breeding parameters, or pest‐prey dynamics. For example, lifecycle data on parasitoids like *Trichogramma* spp or predators such as *C. carnea* are often recorded in paper logs or fragmented research notes, rather than centralized databases suitable for ML. Key limitations include:
  - Temporal gaps: Digital records often span only 1–3 years, whereas AI models typically require 5–10 years of continuous, annotated data for robust training and forecasting.
  - Resolution variability: Data are often recorded weekly or monthly rather than at the finer granularity (*e.g*., hourly or daily) needed to detect micro‐patterns in reproduction, behavior, or stress response.
  - Data type limitations: Available datasets are mostly aggregated and quantitative (*e.g*., total emergence counts), with limited availability of images, videos, or sensor‐rich data needed for behavior tracking, development staging, or anomaly detection.
  - Species representation: Digital records are available for fewer than 10% of commercial NEs species. Most research focuses on well‐known organisms such as *Trichogramma spp*., *C. carnea*, and *Encarsia formosa*, while emerging beneficial species lack digitized rearing data.
  - Lack of integration: Data are often siloed across institutions or projects, with limited centralized databases or standardized data‐sharing protocols. This restricts the development of AI models that can generalize across facilities, species, and environmental conditions. Overcoming these limitations will require digitization at the source, adoption of IoT‐integrated monitoring tools, and the creation of collaborative data‐sharing platforms for BC stakeholders.
- Sensor and imaging constraints: High‐resolution cameras and AI‐integrated sensors can monitor NEs development, reproduction, and predatory behavior. However, such systems are expensive ($10 000–$50 000 per unit) and require specialized expertise for deployment and maintenance limiting their use in resource‐constrained environments.

To address current data limitations, there is an urgent need to develop a centralized, standardized, and accessible data infrastructure specifically for BC. We recommend that international BC organizations, such as the IOBC (https://iobc-wprs.org/), collaborate with research institutions and private industry to establish a shared data platform governed by open‐access principles. This platform should incorporate standardized protocols for data collection, annotation, and interoperability. Additionally, policy initiatives at national and regional levels should support the digitization of historical records, incentivize data sharing across public and private sectors, and fund the creation of secure, scalable, and AI‐ready databases. Collaborative frameworks aligned with the FAIR (Findable, Accessible, Interoperable, Reusable) data principles should be adopted to ensure the consistent, high‐quality, and ethical use of BC‐related datasets in AI applications.

### 6.2 High initial investment

The deployment of AI‐driven technologies in NEs mass production involves substantial capital investment. Key areas include:

- AI‐controlled rearing chambers: These feature automated climate controls, robotic feeding systems, and growth‐stage monitoring. A full‐scale automated rearing unit may cost upwards of $150 000.
- Computer vision‐based diagnostic tools: Used to detect developmental abnormalities or suboptimal rearing conditions in real time.
- Autonomous drones and release systems: Deployed for precision NEs dispersal based on AI‐generated pest distribution maps. These systems require integration with GPS, GIS, and ML‐models.

While such investments are feasible for large‐scale commercial operations, they remain prohibitively expensive for most smallholder and medium‐sized farms. Notably, companies such as Koppert Biological Systems (the Netherlands; https://www.koppert.com/; estimated market share of 20–24% in 2024)^4^ and Biobest Group (Belgium; https://www.biobest.com; estimated market share of 16–20% in 2024)^4^ have begun integrating AI technologies for climate optimization, insect quality control, and NEs mass production. Koppert, for instance, has pioneered the use of predatory mites and developed application tools like the Airobug an air‐blowing system that efficiently distributes NEs (https://www.koppert.com/news-information/news/predatory-mite-pioneers-a-journey-of-biocontrol-innovations/). In Asia, companies such as Qingdao Seawin Biotech Group (China; https://www.seawin-bio.com) and firms like Green Path Company (Vietnam; https://vnbis.com/products/companies/green‐path‐viet‐nam‐trading‐and‐import‐export‐joint‐stock‐company‐538293/) are also piloting AI‐assisted NE production, particularly within greenhouse IPM systems. Industry reports suggest Asian companies may be more receptive to AI integration due to lower labor costs and strong government support for digital agriculture initiatives.

### 6.3 Scalability and adaptability issues

While AI technologies have shown promise in optimizing small‐scale NEs production, scaling these systems for broader agricultural use presents several challenges:

- Automation efficiency: AI‐driven automation must maintain precision across millions of individuals, ensuring consistent reproduction, survival, and predator–prey dynamics.
- Genetic variability: Large‐scale production can lead to genetic bottlenecks, which may reduce the adaptability and effectiveness of NEs in diverse agricultural environments.
- Field application complexities: AI‐driven NEs deployment techniques must include diverse agricultural systems, microclimates, and ecological interactions that affect pest control efficacy.

In addition to technological and operational challenges, ecological strategies such as intercropping can enhance the resilience of cropping systems against invasive pests and support the effectiveness of NEs. Understanding the mechanisms of crop yield formation under different agro ecological setups is critical for integrating AI‐enhanced BC within diversified farming systems. Recent research by Wang *et al*.^57^ underscores the role of spatial crop arrangements and functional biodiversity in suppressing pest outbreaks and optimizing yield outcomes in sustainable agriculture frameworks.

### 6.4 Regulatory and ethical issues

Several regulatory and ethical issues are brought up by the introduction of AI in BC:

- Data privacy and ownership: Local and international data protection regulations must be followed while gathering and using agricultural data, especially when AI is used to support NEs production.
- Ecological risks: AI‐optimized NEs strains may interfere with non‐target species or natural predator–prey dynamics, particularly those altered by selective breeding or genetic augmentation.
- Farmer adoption and trust: Stakeholder education, openness, and the growth of end user trust, many of whom may be unaccustomed to these technologies are essential for the successful deployment of AI‐driven BC solutions.

### 6.5 Synergistic approaches bridging traditional and AI‐enhanced BC

While AI offers transformative advantages for the mass production and deployment of NEs, its full potential is best realized when integrated with traditional BC strategies. Classical methods such as field scouting, ecological habitat management, and manual rearing protocols provide essential biological insights, contextual adaptability, and cost‐effective solutions in low‐tech environments. When paired with AI‐based systems, such as computer vision for quality assessment or predictive analytics for release timing, a hybrid model emerges that is both scalable and ecologically resilient. This synergy enables data‐driven optimization while preserving the ecological validity of BC strategies. For instance, recent research of Ullah *et al*.^59^ emphasizes the importance of integrating machine learning with ecological niche modeling to enhance species‐specific conservation and pest suppression efforts. Such approaches leverage AI's predictive capabilities while grounding interventions in robust biological frameworks. Consequently, the future of sustainable pest management likely resides in the coalescence of AI with proven, field‐tested techniques, ensuring both technological advancement and ecological fidelity.

## 7 FUTURE PROSPECTS

Notwithstanding present difficulties, AI‐facilitated mass manufacturing of NEs possesses transformative potential for sustainable pest management. Numerous exciting advancements are expected imminently:

### 7.1 Advanced AI models for NE optimization

- Predictive breeding and raising: By using machine‐learning models that have been trained on real‐time rearing data, NEs survival and effectiveness can be increased by forecasting ideal breeding conditions, mortality rates, and prey availability.
- Genomic selection: Strong strains of parasitoids and predators with improved adaptability and predation efficiency can be found using AI‐powered genomic approaches.
- Quality assessment: By automating quality checks, AI‐enabled computer vision systems may guarantee that only the most promising candidates are chosen for field deployment.

### 7.2 Autonomous rearing and precision release systems

- Automated rearing chambers: By maintaining precise environmental conditions catered to particular NEs, species, robotics and AI‐driven chambers might greatly increase the efficiency of mass manufacturing.
- Drone‐assisted dispersal: Organizations like UAV‐IQ (USA; https://www.uaviq.com/) have been at the forefront of drone‐based BC, allowing for targeted NEs releases in areas impacted by pests, increasing effectiveness and reducing waste.
- IoT‐based monitoring: IoT solutions facilitate adaptive management by providing real‐time feedback on NEs establishment, mobility, and pest suppression.

### 7.3 AI‐integrated decision support systems for BC

- Real‐time monitoring platforms: AI‐driven platforms support continuous monitoring of NEs population dynamics, pest outbreaks, and environmental interactions, allowing farmers to make informed decisions.
- Digital twin technologies: Simulations of NEs performance across diverse landscapes help optimize mass‐rearing protocols prior to large‐scale implementation.

### 7.4 Cost reduction and wider adoption

The potential for cost reduction through AI‐driven NEs production varies depending on the species, technology, and scale. Current case studies and industry reports suggest the following: (1) Labor and energy savings where partial automation has demonstrated cost reductions of 10–20% due to reduced labor demands, energy use, and human error. (2) Full automation potential wherein optimized systems using real‐time environmental control, machine vision sorting, and robotic handling, cost reductions can reach 30–40%.

- Economies of scale: As AI technologies become more accessible, production costs will decrease. For instance, Kinsect (Italy) reported up to 40% cost reduction through automation in BSF production (https://www.renewablematter.eu/en/Startup-Automated-technologies-for-rearing-of-black-soldier-fly-with-Kinsect).
- Competitive protein production: Full Circle Biotechnology (Thailand) has developed automated insect rearing systems that approach cost parity with conventional protein sources like fishmeal, achieving a reported 25–35% reduction in total production costs (https://www.feedstrategy.com/animal-feed-additives-ingredients/alternative-protein/article/15448323/automated-insect-rearing-could-cut-cost-startup-says).
- Mobile applications: AI‐powered mobile apps can offer localized recommendations for NEs release timing, pest suppression strategies, and intervention planning—enhancing adoption in precision agriculture.
- BC context‐specific savings: For NEs species such as *Trichogramma* or *C. carnea*, where labor and precision are significant cost drivers, 15–30% cost savings are considered realistic. Despite high initial costs, break‐even may be achieved within 2–5 years, depending on the scale and level of automation.

Realistic cost reductions from AI‐driven mass production of NEs range from 10% to 40%, depending on system automation, organism type, and scale. Conservative estimates suggest 15–25% savings in well‐managed facilities, while advanced facilities like BSF rearing startups report up to 40% cost reduction through full automation.

### 7.5 Integration with other precision agriculture technologies

- Remote sensing and GIS: Integration with remote sensing, UAVs, and geographic information systems will enable more accurate, data‐driven pest control strategies. Although outside the primary focus on NEs, advancements in AI‐assisted pollinator rearing systems such as those for bumblebees demonstrate the broader applicability of AI across beneficial insect‐based agricultural practices. These systems may offer transferable frameworks for monitoring, environmental control, and scalability relevant to NEs‐based production.
- Blockchain technology: Blockchain can enhance transparency, traceability, and accountability in the production and deployment of AI‐optimized NEs. Xu *et al*.^58^ highlighted blockchain's role in tracking agri‐food data, improving food safety, reducing transaction costs, and ensuring data integrity. Walmart's pork traceability system is an example of blockchain's potential to build consumer trust through end‐to‐end transparency. These principles can be applied to NEs supply chains, ensuring tamper‐proof, real‐time monitoring and improved system reliability.

### 7.6 Government and institutional support

- Research programs: Government‐backed digital agriculture initiatives in China and Southeast Asia are supporting AI‐assisted NEs production. The EU's Horizon 2020 program has also funded projects like Kinsect's automated BSF rearing system (https://www.renewablematter.eu/en/Startup-Automated-technologies-for-rearing-of-black-soldier-fly-with-Kinsect).
- Policy integration: Organizations such as the International Organization for Biological Control (IOBC) promote research, training, and policy development to facilitate AI integration in BC practices (https://www.iobc-global.org/).

A unified policy framework for digitization and open‐access data sharing is essential to unlock the full potential of AI in BC.

## 8 CONCLUSIONS

In summary, we believe that the future of AI in BC is highly promising. As AI technologies become more advanced, accessible, and affordable, their application in NEs production and deployment will expand across diverse agricultural systems. The development of autonomous production and release systems, adaptive management platforms, and data‐driven optimization tools will enhance the scalability, precision, and efficiency of BC. Ultimately, AI‐integrated NEs production has the potential to revolutionize pest management, reduce dependence on chemical pesticides, minimize environmental impacts, and improve crop yields. With continued technological innovation, interdisciplinary collaboration, and supportive policy frameworks, AI will play an increasingly critical role in sustainable agriculture and food security particularly in the face of climate change.

## CONFLICT OF INTEREST

The authors declare no competing interests.

## Data Availability

Data sharing is not applicable to this article as no datasets were generated or analyzed during the current study.
